# Characteristics of soil salt crust formed by mixing calcium chloride with sodium sulfate and the possibility of inhibiting wind-sand flow

**DOI:** 10.1038/s41598-021-89151-1

**Published:** 2021-05-07

**Authors:** Shenghui Li, Chengzhi Li, Xiaolei Fu

**Affiliations:** 1 Institute of Arid Ecology and Environment, Xinjiang University, Urumqi, 830046 China; 2 College of Resources and Environmental Sciences, Xinjiang University, Urumqi, 830046 China; 3 Key Laboratory of Oasis Ecology Ministry of Education, Xinjiang University, Urumqi, 830046 China

**Keywords:** Environmental sciences, Solid Earth sciences

## Abstract

Soil salt crust can change the structure of aeolian soil and improve its resistance to wind erosion. Four ions (Na^+^, Ca^2+^, Cl^−^, and SO_4_^2−^) with high contents in aeolian soil were selected for a salt crust experiment. The experiment set a variety of gradients of soil salt contents and salt mixing ratios of Na_2_SO_4_ and CaCl_2_. The physical properties of the salt crust were tested, and the wind erosion resistance of the salt crust was discussed. The results showed that the soil salt contents and salt mixing ratio influenced the resistance of the salt crust, especially in terms of its compressive strength and toughness. The former affected the compressive strength of the salt crust by changing the amount of cemented soil salt. The latter affected the kinds of crystals by changing the ion ratio, thus changing the structure of the salt crust and affecting its wind erosion resistance. The wind erosion resistance of the salt crust is complicated by the interaction between the soil salt content and salt mixing ratio. A multilayer crust can be formed in mixed salt, which has a strong wind erosion resistance. This result provides new findings on flowing sand soil and a new method for the treatment of flowing sand soil.

## Introduction

The loose structure of aeolian soil is easily eroded by wind and forms sandstorms^1^, which seriously harm social and economic development and must be addressed. A soil salt crust (SSC) is a cementing layer of soil and salt that forms on the surface or interior of soil when salt crystals bond with soil particles^2^. A salt crust can change the physical and chemical properties of soil and improves soil resistance to wind erosion. Salt crusts mainly occur in arid and semiarid regions with intense evaporation and little precipitation^3–6^. Dry salt lake beds containing a large amount of clay and silt are considered to be the main dust source. However, some lake beds are protected by salt crust, thus greatly reducing dust emission^7^. Significantly, a salt crust can enhance soil resistance to wind erosion in desert areas where vegetation is scarce^3,8–11^. The role of salt crust in sand fixation has gradually attracted the attention of researchers.

The formation of salt crust is controlled by many factors. The ion transport mode in soil is an important factor affecting crust formation^12^. The migration mode of ions affects the precipitation location of crystals in the soil. There are two types of salt crust (efflorescence and subflorescence) that depend on where the salt crystals precipitate^12^. Convection drives the ions to the evaporation surface, while diffusion drives the ions to distribute uniformly in space. When the extent of convection is greater than that of diffusion, salt accumulating on the surface of the medium is named efflorescence, and salt crystallizing in the pores is named subflorescence^13^. There are two types of efflorescence according to the shape of the surface: patchy and crusty^14^. The size of the soil particles affects the salt crust morphology of efflorescence. In coarse soil particles, the rate of evaporation in soil is low, and the sodium chloride crystals formed on the soil surface are large, precipitating on some specific areas of the surface of the substrate and forming a patchy salt crust.

In fine soil particles, the liquid evaporates quickly, and the sodium chloride crystals are small, forming a salt crust that is uniformly distributed on the soil surface^12–14^. Salt crust is also affected by the kind of salt. Salt crust cemented by NaCl usually forms efflorescence at room temperature, while Na_2_SO_4_ usually forms subflorescence under the soil surface, and salt crusts formed by different types of sulfates have different characteristics^15–18^.

Soil salt crust can greatly enhance the wind erosion resistance of soil. The thickness of the salt crust and the mode of soil salt cementation clearly influence the wind erosion resistance of soil^3,19–21^. Increasing the thickness of the salt crust can enhance the stability of the soil surface, thus enhancing its resistance to wind erosion. The mode of soil salt cementing affects adhesion between soil particles; the stronger the adhesion between soil particles is, the stronger the wind erosion resistance^22^. At present, it is clearly understood that the salt species and the characteristics of porous media influence the salt crust. However, the properties of salt crust formed by mixed salts (multiple salts) and its resistance to wind erosion are unclear, so this needs to be addressed.

In nature, salt crust forms mainly by the combination of a variety of salts and soil. Therefore, it is necessary to understand the characteristics of the salt crust cemented by mixed salts. Due to the high content of Na^+^, Ca^2+^, Cl^−^ and SO_4_^2−^ in the aeolian soil in western China, sodium sulfate and calcium chloride were selected for this study. Seven soil salt content gradients and eleven salt mixing ratios were set in a salt crust experiment. The surface morphology, compressive strength and stress-penetration depth curve of the mixture of salt crust were used to explore the influence of the salt mixture ratio and salt content on the properties of the salt crust and to analyze the feasibility of using mixed salt crust to control sand soil. This paper provides a new idea and method for the treatment of flowing sand soil.

## Results

### Surface morphology of salt crust

During water evaporation, ions migrate from the pores of sand to the evaporation front, and the salt concentration on the evaporation surface increases continuously. When the salt concentration reaches supersaturation, the salt crystals precipitate^23^, and the precipitated salt crystals bond with aeolian soil to form a salt crust.

**Figure 1:**
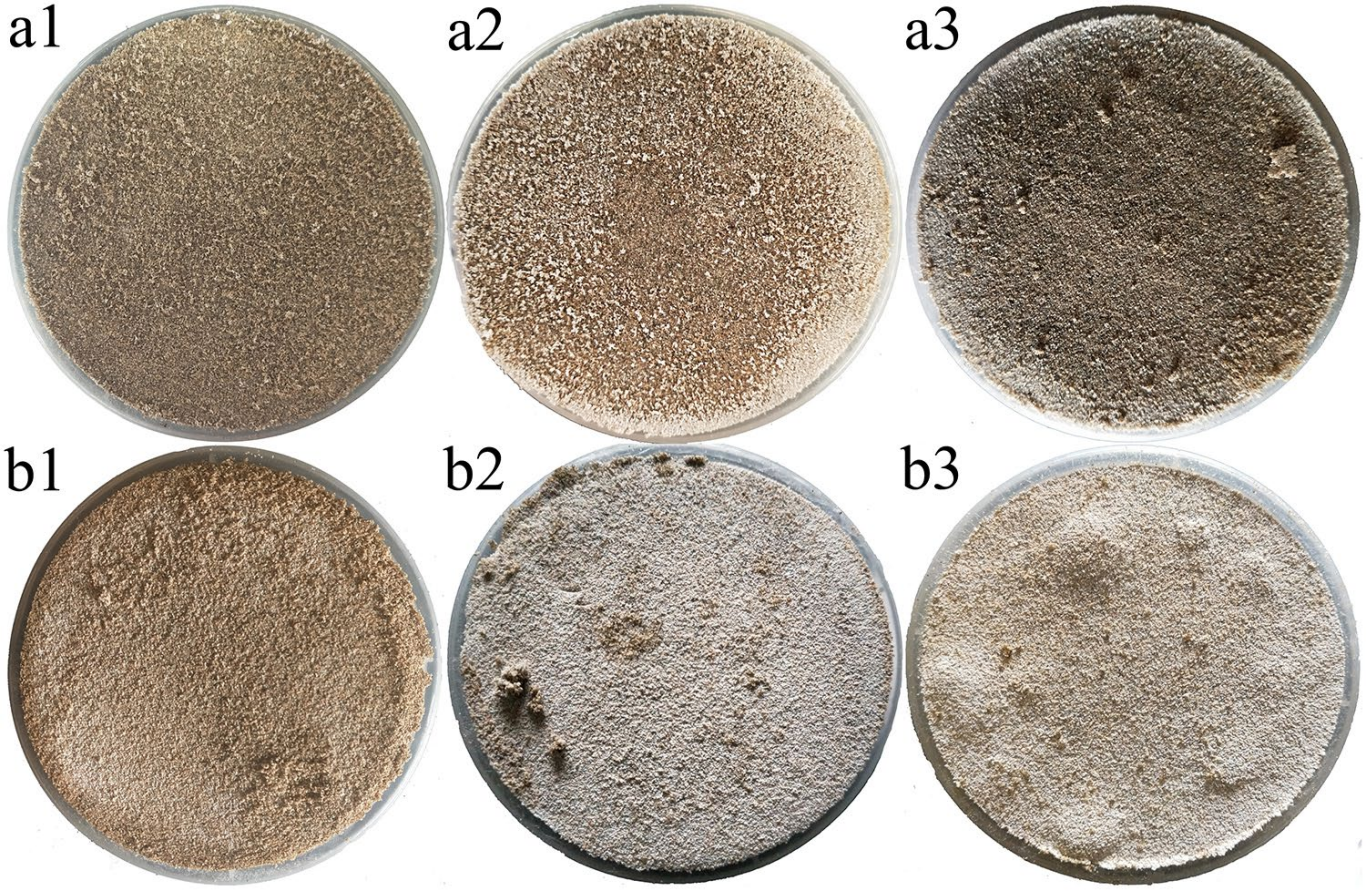
Images of salt crust formed by sodium sulfate or calcium chloride: (**a**) denotes sodium sulfate salt and (**b**) denotes calcium chloride salt. Numbers 1, 2 and 3 represent soil salt contents of 2%, 4% and 6%, respectively. For example, a1 represents the sodium sulfate salt crust with a salt content of 2%.

Figure 1 shows the surface characteristics of a crust of sodium sulfate and calcium chloride. As shown in Fig. 1, with an increase in the salt content in the soil, the surface color of the crust formed by sodium sulfate and calcium chloride single salts gradually deepens, and the number of salt crystals precipitated on the surface increases. When the salt content of sodium sulfate is 2%, the surface of the samples is basically the original color of sandy soil, and a small number of grayish-white mirabilite crystals are scattered on the surface. When the content of sodium sulfate is 4%, the surface color of the samples is gray, and the number of salt crystals on the surface increases, showing a speckled distribution. When the content of sodium sulfate is 6%, the surface color of the samples is close to black and brown, and the crystals are clustered on the surface of the sample.

For the crust formed by calcium chloride, with an increase in calcium chloride content, the surface of the samples is gradually covered by salt crystals of calcium chloride, and the surface color of the crust changes from grayish-white to white. When the content of calcium chloride is 6%, a small number of bumps form on the surface of the sample, and the bumps are not uniformly distributed.

**Figure 2:**
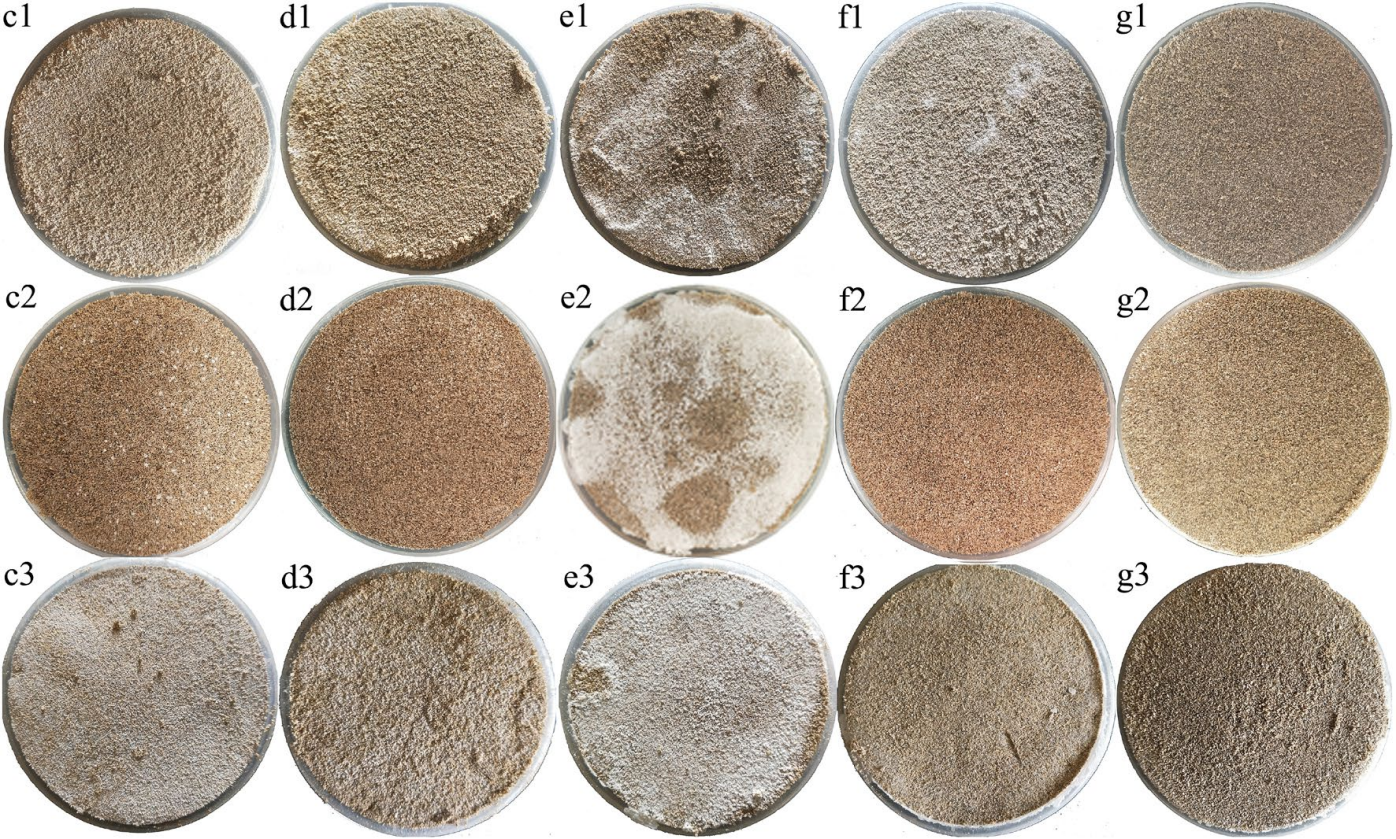
Crust images of salt mixtures composed of sodium sulfate and calcium chloride. Letters indicate the ratio of salt mixture, where (**c**–**g**) indicate salt mixture ratios (sodium sulfate:calcium chloride) of 2:8, 4:6, 5:5, 6:4 and 8:2, respectively. Numbers represent the mass percent of salt in soil, where 1, 2 and 3 represent salt mass percentages of 2%, 4% and 6%, respectively.

The crust formed by a salt mixture is different from that formed by a single salt, and its apparent morphology is more complex. There are three types of salt crust formed by mixed salts. One is an annular or half-moon shape, as c1 and d1 show Fig. 2. The crystals are mostly distributed on the edge and appear annular. In Fig. 2, the salt crystals of d2 are gathered on the surface formed crescent patches. In Fig. 2, f3 also shows a circular crystal scar on the surface. The second type of salt crust presents an uneven speckle distribution or a flower speckle distribution. As shown in Fig. 2, the surfaces of e1 and f1 are flower speckles, and the crystals on the surface are irregularly flaky. In Fig. 2, the salt crystals on the surface of c2 show an uneven dot distribution. The third type of salt is uniformly distributed on the surface, as shown in Fig. 2:c3,d3,e3,g1,g2,g3.

The salt content and ratio of the salt mixture have a great influence on the morphology of the salt crust. At the same salt content, with an increase in the ratio of sodium sulfate, the surface color of the salt crust is first white and then gradually darkens. On line 1 in Fig. 2, the salt content is 2%. When the ratio is 2:8, the surface color of the crust is lighte, and fewer salt crystals precipitate on the surface. With an increase in the ratio of sodium sulfate, the color of the sample surface gradually becomes white, and the extent of salt crystal precipitation gradually increases. When the ratio is 5:5, the surface of the salt mixture crust is the whitest, and the extent of salt crystal precipitation is greates. When the ratio exceeds 5:5, the color of the salt mixture crust darkens bit by bit, and the crystals precipitated on the surface disappear step by step. The contents of the other salts also appear to follow the same law. At the same salt mixture ratio, the number of salt crystals precipitated on the surface increases with an increase in the salt content. From column 3 in Fig. 2, when the ratio is 5:5, the color of the crust surface gradually becomes white with increasing salt content, and the amount of salt crystals precipitated on the surface gradually increases with increasing salt content. However, the law is not obvious for the other columns in Fig. 2.

### Compressive strength

The compressive strength of the salt crust can indicate the wind erosion resistance of soil to a certain extent^23^. The higher the compressive strength is, the stronger the wind erosion resistance^24^.

**Figure 3:**
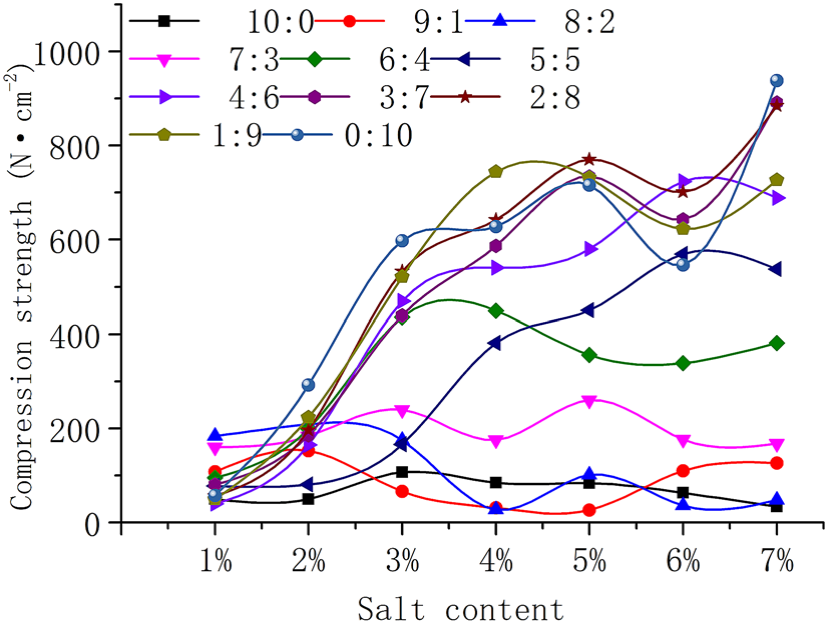
Compressive strength of salt crust with various salt contents.

Figure 3 shows that there are two kinds of crust compression curves. One curve shows that the variation in compressive strength with increasing salt content is small when the salt mixture ratio is 10:0, 9:1, 8:2, and 7:3. The compressive strength fluctuates in the range of 0–250 N‧cm^−2^. For the other curve, the compressive strength increases with the increase in salt content when the salt mixture ratio is 5:5, 4:6, 3:7, 2:8, 1:9, and 0:10.

The proportion of sodium sulfate in a mixed salt has a great influence on the compressive strength of salt crust. The salt content is no more than 2%, sodium sulfate has little influence on the strength of the mixed salt crust, and the change in the strength of the mixed salt crust is small. The content of salt is more than 3%. With a decrease in the ratio of sodium sulfate, the compressive strength of the mixed salt crust gradually increases, and the strength of the salt crust increases gradually. The average values of compressive strength are 67.26, 88.40, 111.1, 194.82, 322.06, 323.11, 457.99, 58.71, 540.25, 516.74, and 539.14 N‧cm^−2^ when the salt ratios are 10:0, 9:1, 8:2, 7:3, 6:4, 5:5, 4:6, 3:7, 2:8, 1:9 and 0:10, respectively. The changes in the compressive strength are 25.22, 48.31, 76.85, 38.77, 129.61, 212.03, 260.241, 292.90, 305.75, 276.27, and 287.2.

### Salt crust stress-penetration depth curve

A manometer can be used to obtain not only the compressive strength (maximum stress) but also the stress-penetration depth curve of the salt crust. The curve can reflect the mechanical characteristics of the salt crust. A manometer was fixed on a pressure workbench. A downward axial force was applied to the crust with a constant speed by the probe of the manometer, and the force measured by the manometer varied with penetration depth. As soon as the probe of the manometer contacted the salt crust surface, the HP software of the manometer began to draw the stress-penetration depth curve. When the first peak of the curve was reached, the maximum shape variable of the salt crust was reached, followed by breakage of the salt crust. The slope of the curve from the onset to the first peak can reflect the toughness of the salt crust. The larger the slope is, the lower the salt crust toughness. The smaller the deformation of the salt crust is when it is broken, the more brittle the salt crust, and the weaker anti-erosion ability of the salt crust. By contrast, the smaller the slope is, the greater the toughness of the salt crust. The better the ductility of the salt crust is, the stronger the anti-erosion ability of the salt crust.

**Figure 4:**
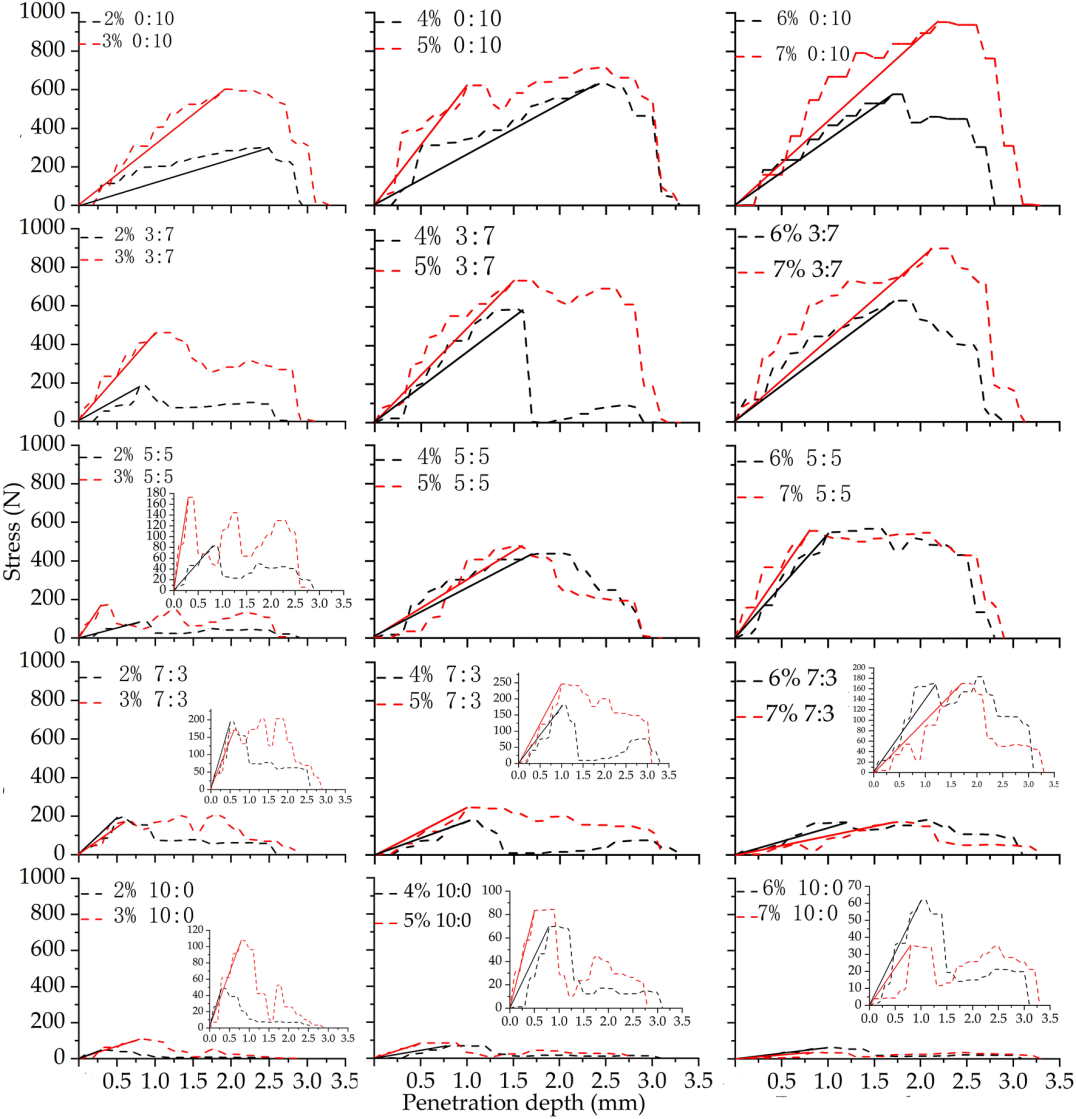
Curves of stress and penetration depth. Percentage represents the salt content, and the proportion represents ratio of sodium sulfate to calcium chloride. For example, 2% 0:10 indicates that the salt content of the sample is 2% and that the mixed salt ratio (sodium sulfate:calcium chloride) is 0:10.

Figure 4 shows the curve of some crust samples. To intuitively reflect the toughness. this paper unified the horizontal and vertical axes of the curve. The longitudinal axes of some inset curves are enlarged in Fig. 4 to intuitively reflect the variation trend of toughness.

As shown in Fig. 4, when the mixed salt ratios are the same, the crust toughness changes with the change in salt content. The influence of changes in salt content on crust toughness can be divided into two parts. In one part, the crust toughness decreases with increasing salt content. For example, when the mixed salt ratio is 0:10, 3:7 and 5:5, the slope of the curve increases with increasing salt content, and the crustal toughness gradually decreases. In the second part, the crust toughness fluctuates slightly in a certain range with increasing salt content. For example, when the salt mixture ratio is 7:3 or 10:0, the slope of the curve decreases first, increases, and then decreases with increasing salt content.

In Fig. 4, the slope of the salt crust curve increase first and then decrease with an increase in the ratio of sodium sulfate at the same salt content. When the salt content is 2%, and the salt mixture ratio gradually changes from 0:10 to 5:5 the slope of the curve gradually increases, i.e., the crustal toughness gradually decreases. When the mixed salt ratio changes from 5:5 to 10:0, the slope of the curve increases gradually, i.e., the crustal toughness increases gradually. The slopes of the curves for the other salt contents also increase first and then decrease with an increase in the ratio of sodium sulfate, but the points at wich the curves change from increasing to decreasing are different. These inflection points observed for 3%, 4% and 5% salt contents occur at 3:7, and the inflection points observed for 6% and 7% salt contents occur at 5:5.

The number of salt crust layers can be determined from the number of peaks in a curve. A single peak means that the salt crust contains a single layer. If more than one peak is observed in the curve, the salt crust contains multiple layers. Figure 4 shows that when the mixed salt ratio is 0:10, the stress-penetration depth curve of the crust is single-peak curves. When the salt ratios are 3:7, 5:5, 7:3 and 10:0, the curve of the salt crust has more than one peak. Therefore, it can be concluded that the crust formed by calcium chloride is mostly a single-layer crust, while the crust formed by sodium sulfate alone or mixed salts is mostly a multilayer crust.

## Discussion

### The effect of mixed salts on crust morphology

The type of crystalline salt is an important factor that influences the apparent morphology of salt crust^25,26^_._ From the finding in “The effect of mixed salts on crust morphology” indicated that the number of salt crystals precipitated on the surface of the sample with sodium sulfate or calcium chloride increases as the salt content increases. The salt crystals on the surface of the mixed salt crust become more complicated as the salt content and salt mixture ratio change. The apparent morphologies of the mixed salt crust and the single salt crust are significantly different, which may be influenced by crystallization products.

The following chemical reaction may occur during the crystallization of a mixed salt composed of sodium sulfate and calcium chloride:

1 $${\text{Na}}_{{2}} {\text{SO}}_{{4}} + {\text{CaCl}}_{{2}} = {\text{CaSO}}_{{4}} + {\text{2NaCl}},$$

**Figure 5:**
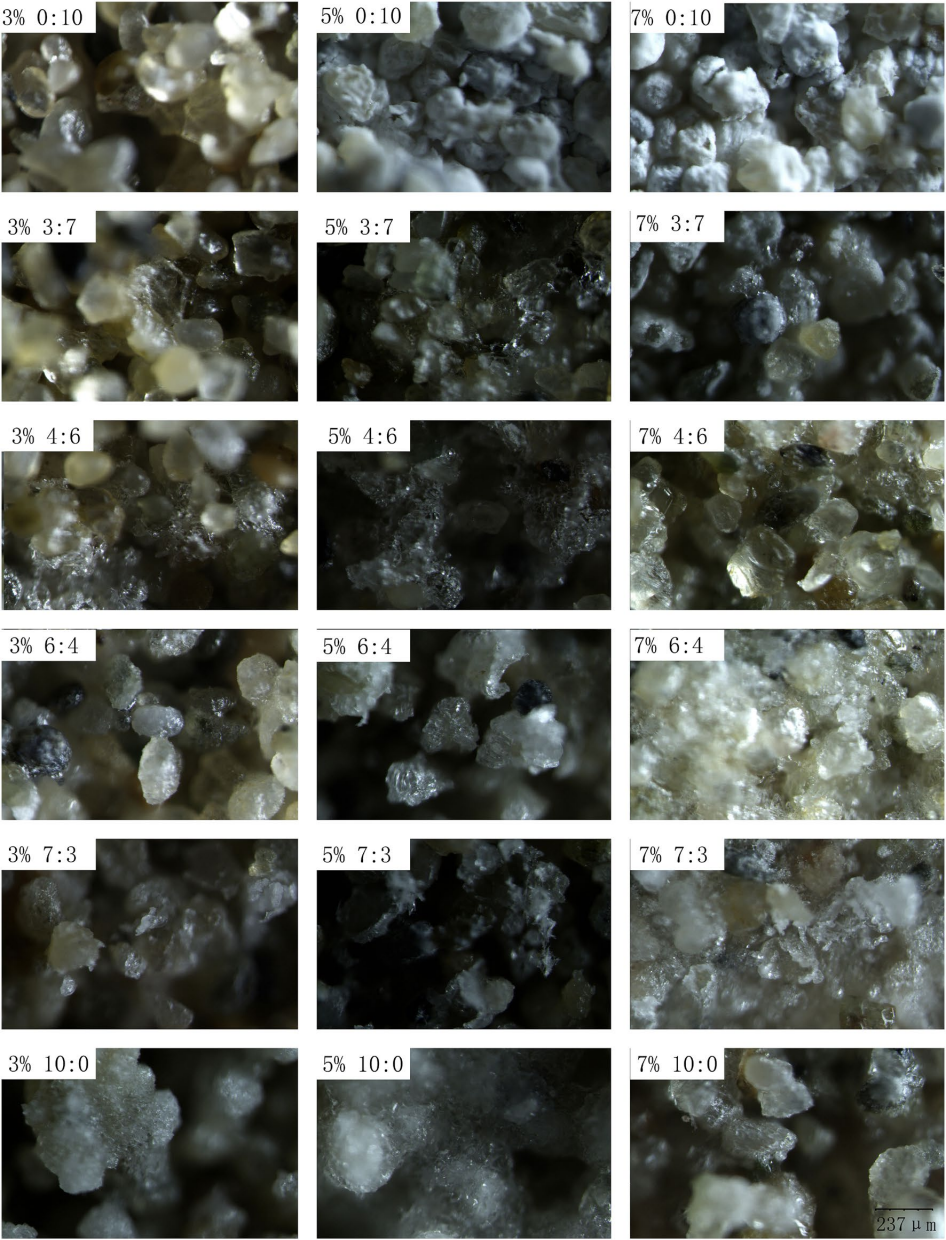
Microscopic images of soil salt crust. An SZ-12 stereomicroscope was used to obtain microscopic images. The percentages represent the mass percentage of salt, and the proportion represents the ratio of sodium sulfate to calcium chloride. For example, 3% 0:10 represents a salt content of 3% and salt mixture ratio (sodium sulfate:calcium chloride) of 0:10.

The mixed salts may form salt crystals of sodium sulfate, calcium chloride, calcium sulfate and sodium chloride in aeolian soil. To verify the crystallization products and discuss the effect of salt species on the crust morphology, a stereomicroscope is used to obtain microscopic images of the upper surface of the salt crust, as shown in Fig. 5.

Figure 5 shows that the sodium sulfate crystal is transparent. The crystal of sodium sulfate grows on the surface of sandy soil and wraps around the sandy soil. With the growth of crystals, the pores of the soil are filled with salt crystals. On the soil surface, the crystals form a flake structure with a certain texture, and some crystals gather to form larger salt crystal particles. The calcium chloride crystal is a grayish-white small-volume crystal, and it is adsorbed on the surface of sandy soil particles.

When the sand particles are completely coated by calcium chloride crystals, the color of the surface of the sample turns gray-white. The crystals of calcium chloride and sodium sulfate cement aeolian soils in completely different ways. Crystals of calcium chloride are absorbed on the surface of aeolian soil particles, and the particles absorbing the crystals of calcium chloride are still independent. With an increase in the number of calcium chloride crystals on the surface of soil particles, the number of soil salt cementing particles increases continuously, finally causing the particles to aggregate to form a stable soil salt crust. Sodium sulfate crystals crystallize in the pores of sandy soil. The crystals of sodium sulfate aggregate to form large crystals and then wrap around the upper surface of the particles of sandy soil, forming a soil salt crust.

Mixed salts can also produce sodium chloride and calcium sulfate crystals. In Fig. 5, we observed not only sodium sulfate and calcium chloride crystals but also sodium chloride crystals, which are silver-white cubic crystals. Desarnaud^28^ studied the nucleation and growth of sodium chloride crystals from a molecular point of view and found that sodium chloride crystals grew on the surface of polyvinylidene fluoride (PVDF) and formed a cubic lattice structure. According to the crystal morphology in Fig. 5 and Eq. (1) in the paper, the crystals can be determined to be sodium chloride crystals^9,28–30^. However, from Fig. 5, calcium sulfate crystals are not observed on the upper surface of the sample, as they may crystallize in the interior of the soil.

From the apparent morphology of the mixed salt crust in Fig. 2, the salt crystals of some samples are deposited on the edge of the sample surface. The salt crystals of some samples are irregularly distributed on the surface of the samples, forming heterogeneous patches. Salt crystals of some samples are uniformly distributed on the surface of the samples. Mina et al. studied the crystallization of sodium chloride in porous media and found that sodium chloride tends to crystallize at the edge of the matrix^31^. This is because the evaporation rate is higher at the edge of the sample than in the middle and the solution at the edge of the sample is more likely to reach saturation, precipitating more crystals.

The particle size and properties of soil affect the location of crystal precipitation, and crystals tend to crystallize on the surface of hydrophilic media^32^. In this paper, the particle size of sandy soil is not uniform, and there are many kinds of minerals in sandy soil. The formation of heterogeneous speckles on the upper surface of some samples may be caused by the heterogeneity of sandy soil. Qazi studied the evaporation of sodium chloride solution in porous media and found that the higher the salt concentration is, the faster the crystal precipitation and the more uniform the crystals distributed on the surface of the medium^33^. In this paper, the apparent morphology of the crust and the distribution of salt crystals on the surface are consistent with the law described by Qazi.

Moreover, the location of crystal precipitation and crystal morphology were affected by relative humidity (RH). Carlos reported that the crystallization pattern of sodium sulphate was strongly affected by RH, while the RH effect on sodium chloride was less. Prismatic acicular or bulky thenardite crystals appear at RHs less than 50%, while crystallization at 20% RH resulted in the formation of either euhedral or rhombohedral thenardite crystals^34^. The change in RH will have an impact on the crystal morphology of sodium sulphate, and the RH of the air in desert in western China changes with time; thus, the RH in the experiment was not controlled deliberately, and it changed with ambient conditions during the experiment. The impact of RH change on the experimental results needs to be further investigated.

### Effect of mixed salts on the compressive strength of crust

Cementation between salt crystals and soil particles changes the loose structure of sandy soil, thus enhancing the compressive strength of sandy soil. From “Compressive strength”, it can be seen that the compressive strength of sodium sulfate soil salt crust changes little with increasing salt content. The compressive strength of the calcium chloride salt crust increases with increasing salt content. In the mixed salt, when the proportion of sodium sulfate is large, there are more sodium sulfate crystals in the salt crust, the compressive strength of the salt crust is similar to that of the sodium sulfate salt crust, and the salt crust strength fluctuates slightly with changes in salt content. When the proportion of sodium sulfate in the mixed salt is small, there are more calcium chloride crystals in the salt crust, and the compressive strength of the salt crust is similar to that of the calcium chloride salt crust.

The compressive strength of salt crust increases with the increasing mass percentage of salt. This phenomenon may be related to the cementation mode of salt crystals. Figure 5 shows that the crust formed by sodium sulfate is mainly a lamellar structure with texture, which is easily broken when it is subjected to vertical pressure or high-speed erosion of sand flow. Calcium chloride crystals directly adsorb on the surface of sandy soil particles and fill in the pores between sandy soil particles to increase the internal friction between sandy soil particles and gradually forming a consolidated soil salt. This structure is relatively stable and has high compressive strength.

The salt crust formed by mixed salts includes not only sodium sulfate crystals and calcium chloride crystals but also sodium chloride crystals and calcium sulfate crystals. From Fig. 5, with the mixing ratio approaching 5:5, more sodium chloride crystals can be observed on the crust surface. Many sodium chloride crystals can be seen in Fig. 5 (7% 6:4) and (7% 4:6). The existence of sodium chloride and calcium sulfate crystals complicates the change in the compressive strength of the mixed salt crust with changing the salt mixture ratio and salt content. In Fig. 3, the content of salt is no more than 2%, and there is a small difference between the compressive strengths of the salt crust with a variety of mixed salt ratios.

As the number of crystals in this salt crust is low, the stability of the salt crust is poor. The bonding mode between salt crystals and sandy soil particles has a small influence on the compressive strength. When the salt content is higher than 3%, the number of crystals is greater, and the bonding mode between salt crystals and sandy soil particles could influence the compressive strength. With an increase in the salt content, the number of salt crystals increases continuously, the salt crust thickness increases, and the compressive strength increases accordingly. However, the compressive strength does not increase linearly with an increase in salt content because the salt mixing ratios affect the crystallization products and the boding mode between salt crystals and sandy soil particles, which affects the mechanical characteristics of the salt crust.

**Figure 6:**
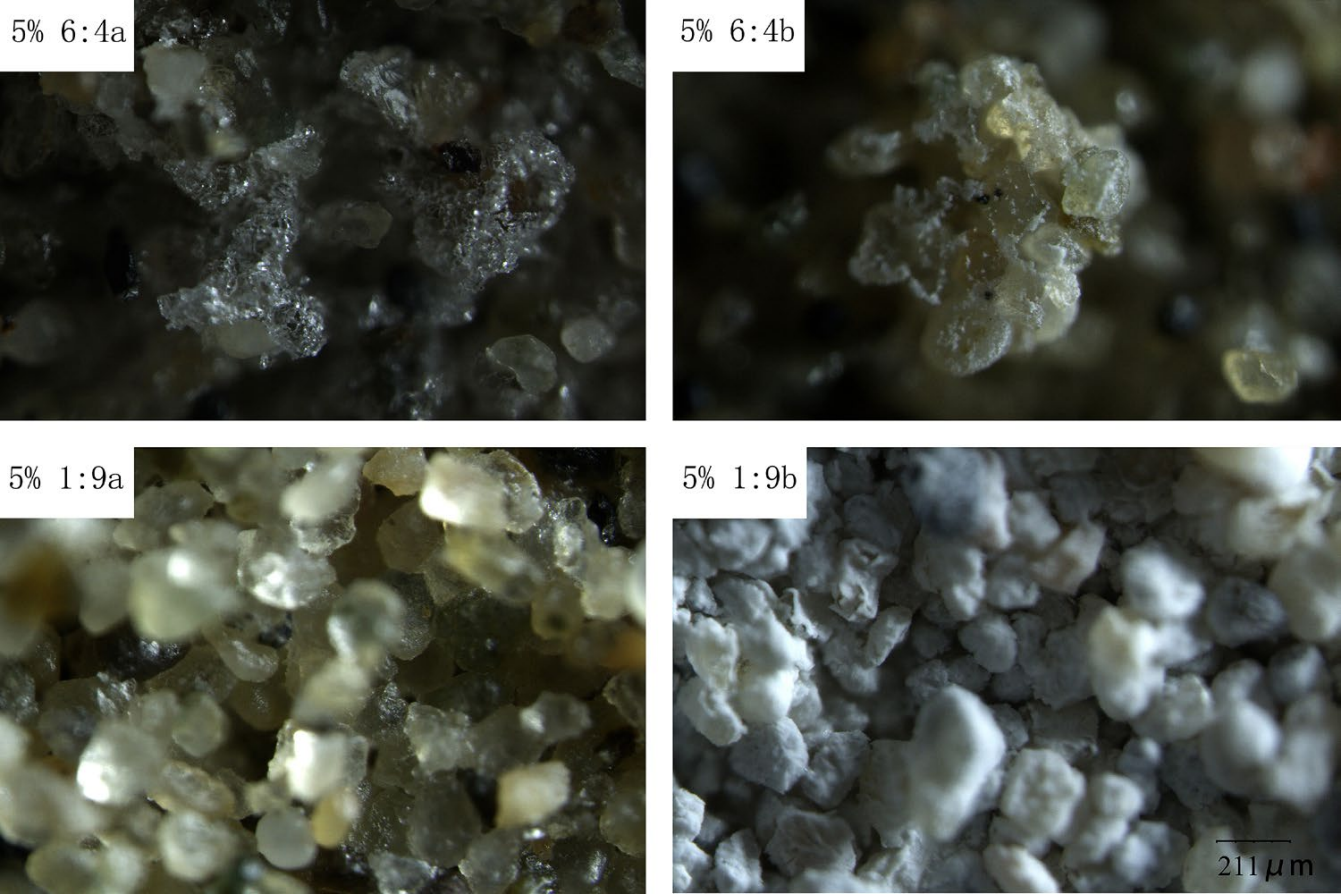
Comparison of microstructure images at different locations of the same sample surface. Salt-crust samples with a 5% mass percentage of salt and salt mixing ratios of 6:4 and 9:1.

Several points on the surface of the same sample were observed with a stereomicroscope, as shown in Fig. 6. Two kinds of crystalline salts, sodium sulfate (Fig. 6, 5% 6:4a) and calcium chloride (Fig. 6, 5% 6:4b), were found on the salt-crust surface of the mixed salt. On the surface of the sample with a mass percent of 5% and salt mixing ratio of 1:9, sodium sulfate and calcium chloride crystals were also found. Both salts coexist in the mixed salt crust. This can also be confirmed by e1, e2 and f3 in Fig. 2. The heterogeneous aggregation of salt crystals on the surface is affected by the kind of salt and the properties of the media, which may be one of the reasons why the compressive strength and toughness of the salt crust fluctuate greatly.

The mixture of sodium sulfate and calcium chloride produces four crystals: sodium sulfate, calcium chloride, sodium chloride and calcium sulfate. The quantity and spatial distribution of crystals fluctuate greatly with changes in the mixing ratios of salts. This phenomenon complicates the change in the compressive strengths of mixed salts with changes in the salt content and the salt mixture ratio. The mechanism of this change is still unclear, and the effect of mixed salt crust on the compressive strength of mixed salt must be studied further.

### Wind erosion resistance of mixed salt crust

During a wind erosion event, soil particles show three different modes of transport: suspension, saltation, and creep^35^. The wind erosion resistance of salt crust can be considered from three aspects: compressive strength, toughness and the number of salt crust layers.

Salt crystals bind with sandy soil to form salt crust, and the compressive strength of salt crust comes from two sources. One source is the salt crystals encasing soil particles bind with each other to form a whole, thus changing the loose structure of sandy soil and enhancing its compressive strength^26^. The other source is the crystals growing in soil pores to fill the soil pores and increase the friction between particles, thus enhancing the compressive strength of soil^21^. The two kinds of salt crusts change the loose structure of sandy soil, and the compressive strength of the surface layer improves. However, due to the increase in the compressive strength of the salt crust, the first cementing mode has little influence on the compressive strength of the salt crust, and the increase in the compressive strength of the salt crust with an increasing mass percent of salt is also small. The second cementing mode has a great influence on the compressive strength of the salt crust, in which the compressive strength of the salt crust increases rapidly with an increase in the mass percent of salt. From the perspective of soil wind erosion, the second cementing mode is obviously better than the first crystal cementing mode.

Stable salt crust covers the surface of loose sand soil to resist wind erosion^36^. The larger the proportion of salt crust coverage is, the stronger its wind erosion resistance. If the salt crust is broken, the particles jump and accelerate with the wind and finally hit the crust surface, abrading the crusted surfaces in a process similar to sandblasting and increasing the release of dust^37^. This situation can be avoided if there are multiple layers of salt crust. Even though the upper layer of crust ruptures, the lower layer of crust still resists wind erosion, and the soil erosion resistance does not decline sharply. Therefore, multilayer salt crust is helpful in enhancing and maintaining the wind erosion resistance of sandy soil. By analyzing the stress-penetration depth curve of the crust, we find that the salt crust formed by calcium chloride is mostly single-layer crust, and the salt crust formed by the mixed salt is nearly multilayer crust. Therefore, the wind erosion resistance of multilayer crust formed by mixed salts may be better than that of single-layer crust^23^.

The toughness of the salt crust reflects the deformability of the salt crust under stress. The stronger the toughness of the salt crust is, the less erosion of the salt crust that occurs by wind or sand saltation. The stress-penetration depth curve of the salt crust shows that the toughness of sodium sulfate salt crust is better than that of calcium chloride salt crust and mixed salt crust. However, note that the compressive strength of sodium sulfate is low, and its resistance to wind erosion is weak. The mixed salt crust can increase the compressive strength of salt crust; the toughness of mixed salt crust is also better, with a relatively rough surface^32^. Therefore, mixed salt crust is more resistant to wind erosion than single salt crust.

While salt crusts are friable or fractured, salt crusts may be eroded by wind or sand saltation, increasing dust in the atmosphere^36^, thus, the kinds of salts used for cementing sand soil particles should be selected with careful consideration. In this study, the salt crusts of mixed salts is tighter than the loose structure of sandy soil, so this salt crust can inhibit dust emissions. The intraparticle salt bonds of salt crust maybe disappear during heavy rain. Therefore, the application of salt crust to sand soil treatment has certain limitations, so salt crust should be used in arid areas with low rainfall and high evaporation^38^. In the deserts in western China, the annual rainfall is less than 150 mm, and little rainfall within 0–5 mm accounts for approximately 70% of the total rainfall frequency^39–41^. After rain, rapid evaporation can lead to rapid salt crust formation.

## Conclusions

By analyzing and discussing the apparent characteristics, compressive strength, stress-time curve and microscopic morphology of salt crust formed by the mixture of sodium sulfate and calcium chloride salts, the following conclusions were drawn:

1. When there is only one kind of salt crystal, the microstructure of the salt crust is uniform. The compressive strength of salt crust formed by one salt is affected by the number and distribution of crystals. Thus, the compressive strength of salt crust formed by sodium sulfate or calcium chloride salts has a good correlation with salt content in the soil. The compressive strength of mixed salt crust is affected not only by salt content but also by the salt mixture ratio. The microstructure of a mixed salt crust is complex, and the compressive strength of salt crust fluctuates greatly with changes in the salt content and salt mixture ratios. This phenomenon is related to the mode of soil salt cementation and the location of crystal settling.
2. The crystallization products of mixed salts include sodium sulfate, calcium chloride, calcium sulfate and sodium chloride crystals. The number of crystals depends on the proportion of the mixture. The number and type of salt crystals are influenced by the binding sequence and content of Na^+^, Ca^2+^, Cl^−^ and SO_4_^2−^, which affect the apparent morphology and compressive strength of crust, leading to a more complex impact on the compressive strength of mixed salts with various salt contents and salt mixture ratios.
3. Salt crust formed by mixed salts is mainly multilayer crust. Even after the upper salt crust is damaged, the lower salt crust can still protect the soil from erosion, and the wind erosion resistance of the crusted soil is greatly improved. Meanwhile, the toughness of mixed salt crust is higher than that of crust containing one salt, and mixed salt crust can withstand multiple blows without damage and exhibits better durability than single salt crust. A salt crust containing a mixture of sodium sulfate and calcium chloride salts is more suitable for aeolian soil control when the compressive strength, toughness and number of layers in the crust are all considered. To achieve aeolian soil control, the optimal mass percent and optimal salt mixture ratio in soil are 7% and 3:7, respectively.

## Materials and methods

### Experimental materials

The aeolian soil used in the experiment was collected from the upper surface layer (0–20 cm) of a migratory dune in the Gurbantunggut Desert, western China. The sandy soils used for the salt-crust experiment were screened using a shaking sieve in which the mass percent of the 150–300 μm particle size was 13.5%, the mass percent of the 100–150 μm particle size was 68.5%, and the mass percent of other particle sizes was 18%. To eliminate the influence of sand particle size on the experiment, the representative soil particles, selected as 100–150 μm particles, were used for the experiment. Deionized water was used to clean the soil to reduce the influence of a small amount of soluble salt ions left in the sandy soil in the salt crust test, and the washed soil was dried.

### Preparation of salt crust

According to previous salt crust experiments^25,26^, 7 total salt mass percentages and 11 salt mixture ratios (mass ratios of the two salts, namely, sodium sulfate:calcium chloride) were set in this paper. The total salt mass percentages were 1%, 2%, 3%, 4%, 5%, 6% and 7% (salt as a percentage of soil mass, hereinafter referred to as salt content). The 11 salt mixture ratios (sodium sulfate:calcium chloride) were 0:10, 1:9, 2:8, 3:7, 4:6, 5:5, 6:4, 7:3, 8:2, 9:1 and 10:0.

The experiment was conducted at 30 °C. The solubilities of sodium sulfate and sodium chloride at this temperature were 40.8 g and 100 g, respectively. During the production of the sample, the soil mass was fixed at 750 g. The salt mixture was added to the sandy soil, and 100 ml of deionized water was added and stired evenly. Finally, the mixture was placed in a sand mold (a plastic cylinder with a diameter of 5 cm and height of 3.5 cm), and a scraper was used to smooth the surface of the sand mold. The total mass of the sample was controlled to 160 g. Five samples were prepared for each gradient. The highest temperature on the sand surface in July in the northern desert of China is approximately 70 °C^38^. The highest temperature on the sand surface in the Gurbantunggu Desert in China is 84 °C^42^, and the highest temperature in the Batain Jaran Desert in China is ranges 70–80 °C^43^. Considering the surface temperature in the summer in the sampling area and experimental time, the experimental samples were dried at 75 °C in a drying oven. When the sample mass reached a constant weight, it was cooled in a dryer to room temperature. Since the average RH in the Gurbantunggut Desert is 40–50% in the summer, the effect of RH on crust formation is not discussed^44^.

### Data acquisition and processing

A photograph of the sample surface was taken with a digital camera, and the color and apparent form of the salt crust were recorded. The compressive strength of the salt crust was probed with a digital electronic manometer (HP, accuracy: 0.01 N; range: 0–1000 N), A downward axial force was applied to the crust with the probe of the manometer operated at a constant speed (1 mm‧s^−1^). The manometer recorded the force as a function of penetration depth and formed a stress-penetration depth curve. Five samples of each gradient were tested. When the stress value of the stress-penetration depth curve reached the first peak, this value was recorded because it represented the compressive strength of the crust. The mean of five stress values was obtained as the compressive strength of the gradient sample.
